# Physical activity and cardiovascular risk in obese older Korean women: effects on obesity, hypertension, and arterial stiffness

**DOI:** 10.3389/fpubh.2025.1580825

**Published:** 2025-06-11

**Authors:** Woo-Hyeon Son, Yi-Sub Kwak, Min-Seong Ha

**Affiliations:** ^1^Design Institute, Inje University, Gimhae, Republic of Korea; ^2^Department of Physical Education, Dong-Eui University, Busan, Republic of Korea; ^3^Laboratory of Sports Conditioning: Nutrition Biochemistry and Neuroscience, Department of Sport Science, College of Arts and Sports, University of Seoul, Seoul, Republic of Korea

**Keywords:** physical activity, body composition, blood pressure, arterial stiffness, older women, cardiovascular health

## Abstract

**Background:**

With the global aging population, the increasing prevalence of chronic diseases has emerged as a critical public health issue. Obesity, in particular, is a major risk factor for cardiovascular diseases (CVD), such as hypertension and arterial stiffness (AS). Regular physical activity (PA) may help mitigate these risks. This study aimed to investigate the effects of PA levels on body composition, blood pressure (BP), and AS in Korean obese older women.

**Methods:**

A total of 313 obese older women were enrolled and classified into an inactive group (ING, *n* = 160) and an active group (AG, *n* = 153) based on their levels of PA. Body composition was assessed using bioelectrical impedance analysis, BP measured with an automated sphygmomanometer, and AS was evaluated by measuring carotid-femoral pulse wave velocity (cfPWV). Differences between the groups were analyzed using independent sample *t*-tests, and correlation analyses were conducted to examine relationships among key variables.

**Results:**

The active group exhibited significantly lower body fat percentage, systolic blood pressure (SBP), and cfPWV (all *p* < 0.001) compared to the inactive group, while skeletal muscle mass was significantly higher (*p* < 0.05). Furthermore, significant positive correlations were observed between body fat percentage and SBP, as well as between SBP and AS.

**Conclusion:**

These findings demonstrate that regular PA is associated with improved body composition and reductions in both BP and AS in obese older women. The results underscore the importance of promoting PA as a preventive strategy against CVD in the aging population. Further research is warranted to explore the effects of various intensities and types of PA on vascular health and metabolic function.

## Introduction

The global population is aging at an unprecedented rate, with the number of individuals aged 65 and older having doubled since 1974 (1). In South Korea, older adults currently represent 18.4% of the total population, and this proportion is projected to exceed 20.6% by 2025, officially marking the country’s transition into a super-aged society (2). This demographic shift is accompanied by various social and health challenges, among which the increasing prevalence of chronic diseases is particularly prominent (3).

One of the most effective strategies for mitigating age-related health decline is the promotion of physical activity (PA) (4). However, PA levels tend to decrease with age. Globally, approximately 31.3% of individuals aged 60 and above are physically inactive (5), contributing to unfavorable changes in body composition—such as increased fat mass and decreased basal metabolic rate—that heighten the risk of chronic conditions including obesity (6, 7).

A previous study has demonstrated an inverse relationship between PA levels and obesity indicators such as percentage body fat (%BF) and body mass index (BMI) (8). Obesity in older adults has been closely associated with a higher incidence of hypertension and cardiovascular disease (CVD). Excess adiposity contributes to sympathetic nervous system overactivation and stimulates the renin-angiotensin system (9, 10), mechanisms that elevate blood pressure and promote vascular dysfunction (11). Furthermore, obesity-induced metabolic dysregulation and chronic inflammation are known to impair vascular elasticity and contribute to arterial stiffness (AS) (12).

Arterial stiffness, commonly assessed via pulse wave velocity (PWV), is a non-invasive biomarker of vascular aging. Köchli et al. reported that AS increases in parallel with rising BMI and systolic blood pressure (SBP) (13). A 1 m/s increase in central PWV has been linked to a 14% rise in the risk of cardiovascular events (14, 15). Similarly, SBP and BMI are positively correlated with AS, underscoring the intertwined relationships among body composition, blood pressure, and vascular health.

Evidence suggests that regular PA has multifaceted benefits: it increases energy expenditure (16), promotes weight loss, lowers blood pressure (17), and enhances endothelial function through improved nitric oxide (NO) bioavailability (18, 19). These physiological adaptations collectively contribute to a reduction in AS (20). Indeed, previous studies have demonstrated that moderate-intensity PA is inversely associated with both hypertension (21, 22) and PWV, and that a 10 mmHg reduction in SBP may lower cardiovascular event risk by up to 25% (23). Molis et al. further reported a 12.5% improvement in PWV among older women who participated in moderate-intensity PA (24).

Based on these findings, public health authorities such as the Centers for Disease Control and Prevention (CDC) recommend that adults aged 65 years and older engage in at least 150 min of moderate-intensity aerobic activity per week—including exercise, household chores, and leisure-time activities—as a means of preventing obesity and cardiovascular diseases (25).

Although the cardiovascular and metabolic benefits of PA have been well documented, few studies have specifically examined the relationship between PA levels and health outcomes such as body composition, BP, and AS in obese older women in South Korea—a population that is particularly vulnerable to these interrelated cardiovascular and metabolic risk factors.

While numerous studies have investigated the effects of structured exercise interventions in older adults, including older women (26), much less is known about how habitual levels of daily PA, as measured objectively by accelerometry, relate to key cardiometabolic parameters in community-dwelling obese older women. This distinction is critical, as structured exercise and free-living physical activity may have different patterns of association with health indicators in real-world settings. Furthermore, most previous studies have either focused on general older populations or pooled data across sexes, limiting the understanding of PA-related outcomes in high-risk subgroups such as obese older women.

Given the rapid increase in the aging population and the high prevalence of physical inactivity and obesity among older Korean women, a better understanding of how varying levels of habitual PA influence body composition, BP, and AS is warranted. Investigating these relationships may offer novel, population-specific insights that are essential for developing tailored public health strategies and preventive interventions for this at-risk group.

Therefore, the present study aims to investigate the associations between objectively measured physical activity levels and body composition, blood pressure, and arterial stiffness in obese older Korean women. The findings are expected to contribute meaningful evidence toward personalized approaches for promoting healthy aging and preventing cardiovascular complications in this vulnerable population.

## Methods

### Participant characteristics

The study was conducted on obese older women (body fat percentage, %BF ≥ 35%) (27) aged 65 years and older living in Busan Metropolitan City. A total of 311 participants were recruited and divided into two groups: an inactive group (ING; *n* = 160) and active group (AG; *n* = 153).

All eligible participants had no history of mental or neurological disorders and were not undergoing hormone therapy. Additionally, individuals with a history of cardiovascular disease or diabetes were excluded. Participants had no prior experience with similar experimental procedures and had not made any significant changes to their usual physical activity levels in the past 6 months. All participants voluntarily agreed to participate in the study and provided written informed consent approved by the Institutional Review Board of the University of Seoul. All protocols were approved by the Institutional Review Board (IRB No: UOS 2024-09-009-003) of the Public Institutional Review Board designated by the Department of Health and Human Services. The study was conducted in accordance with the Declaration of Helsinki, and written informed consent was obtained from all participants prior to the study. The general characteristics of the participants are shown in Table 1.

**Table 1:** Characteristics of participants.

Variables	ING	AG
Age (years)	75.25 ± 4.30	74.21 ± 4.69
Wear time (min/day)	785.13 ± 67.27	788.03 ± 65.35
Sedentary (min/day)	510.21 ± 72.93	471.64 ± 63.21
LPA (min/day)	312.17 ± 84.78	285.26 ± 72.65
MVPA (min/day)	5.28 ± 3.37	42.54 ± 15.39

Values are presented as mean (M) ± standard deviation (SD). *p < 0.05, ***p < 0.001.

LPA, low physical activity; MVPA, moderate to vigorous physical activity; ING, inactive group; AG, active group.

### Accelerometry

The participants’ PA (classified as light, moderate, or vigorous) and sedentary time were assessed using an ActiGraph^®^ GT1M accelerometer (Fort Walton Beach, FL, United States). This device is a valid tool for objectively measuring the frequency, duration, and intensity of PA (28). It can also be utilized to assess periods of inactivity by recording low-amplitude values (29). A valid measurement day was defined as one that met 600 min (10 h) of wear time per day (30). Only participants with at least three valid days of data (including 2 days during the week and 1 day on the weekend) were included in the analysis.

The intensity of PA was stratified into the following categories based on the count value recorded from the accelerometer. Sedentary activity was defined as less than 100 counts/min, light PA as 100–2019 counts/min, moderate PA as 2,020–5,998 counts/min, and vigorous PA as 5,999 counts/min or higher (31). Accordingly, the frequency, duration, and intensity of PA were analyzed, and the participants’ PA levels were assessed according to the World Health Organization’s Global Recommendations on Physical Activity for Health (32). Participants were considered physically active if they averaged at least 21.4 min of moderate-to-vigorous PA per day, and inactive if they did not.

### Anthropometrics and body composition

The height, weight, and body composition of the participants were measured while they were wearing light clothing. Height was measured in 0.1 cm increments employing a portable stadiometer (InLabS50, Inbody, Korea). The body weight, %BF, and skeletal muscle mass (SMM) were assessed using bioelectrical impedance analysis (InBody, Korea) (33).

The body mass index was calculated using the following equation.

$\mathrm{BMI}=\text{weight}\;(\mathrm{kg})/\text{height}\;(m^{2})$ .

### Blood pressure

Brachial artery BP was measured in the left upper arm using an automated sphygmomanometer HEM-7156 T (Omron, Japan) after 5 min of rest in a seated position during a visit to the laboratory. Two measurements were obtained, with a two-minute interval between them, and the mean value was used for the analysis. If the discrepancy between BP readings exceeded 10 mmHg, additional measurements were obtained.

### Arterial stiffness

Patients arrived at the laboratory 20 min prior to measurement, and were asked to recline and rest for approximately 10 min. AS was measured using the Sphygmocor Xcel cuff device (AtCor Medical, CardieX, Sydney, Australia), a tonometer-based pulse wave measurement device that noninvasively detects pressure pulse waves. The measurements were analyzed using the Sphygmocor Cardiovascular Management Suite analysis software (version 8.0). PWV was calculated by dividing the distance (D) between the carotid and femoral arteries by the difference (t) in pulse wave transit times (PWTTs) between the two arteries to obtain the velocity (34). Two measurements were taken, and the average value was used.

The equation is as follows.

$\begin{array}{l} \mathrm{PWV}\;(m/s)=\text{Distance between the}\;\mathrm{two}\;\text{arteries}\;(D)÷\\ \;\text{PWTT}\;(\Delta t) \end{array}$ .

### Statistical analysis

The required sample size was calculated using the G*Power version 3.1 software for Windows (Kiel University, Kiel, Germany), based on a default effect size of 0.3, an alpha level of 0.05, and a statistical power of 95% (35). As a result, the estimated number of participants required for the study was 138. Considering a 25% dropout rate and potential missing data, the final sample size was set at 330 participants. All statistical analyses were performed using the SPSS statistical program 25.0 package (SPSS Inc., Chicago, IL, United States). All data are expressed as mean ± standard deviation. Independent sample *t*-tests were performed to ascertain the differences in the BP and AS among the PA groups. Correlation analyses were used to elucidate the relationship of PA with BP and AS. Statistical significance was set at ≤ 0.05.

## Results

### Correlations between obesity, body composition, blood pressure, and arterial stiffness

**Figure 1:**
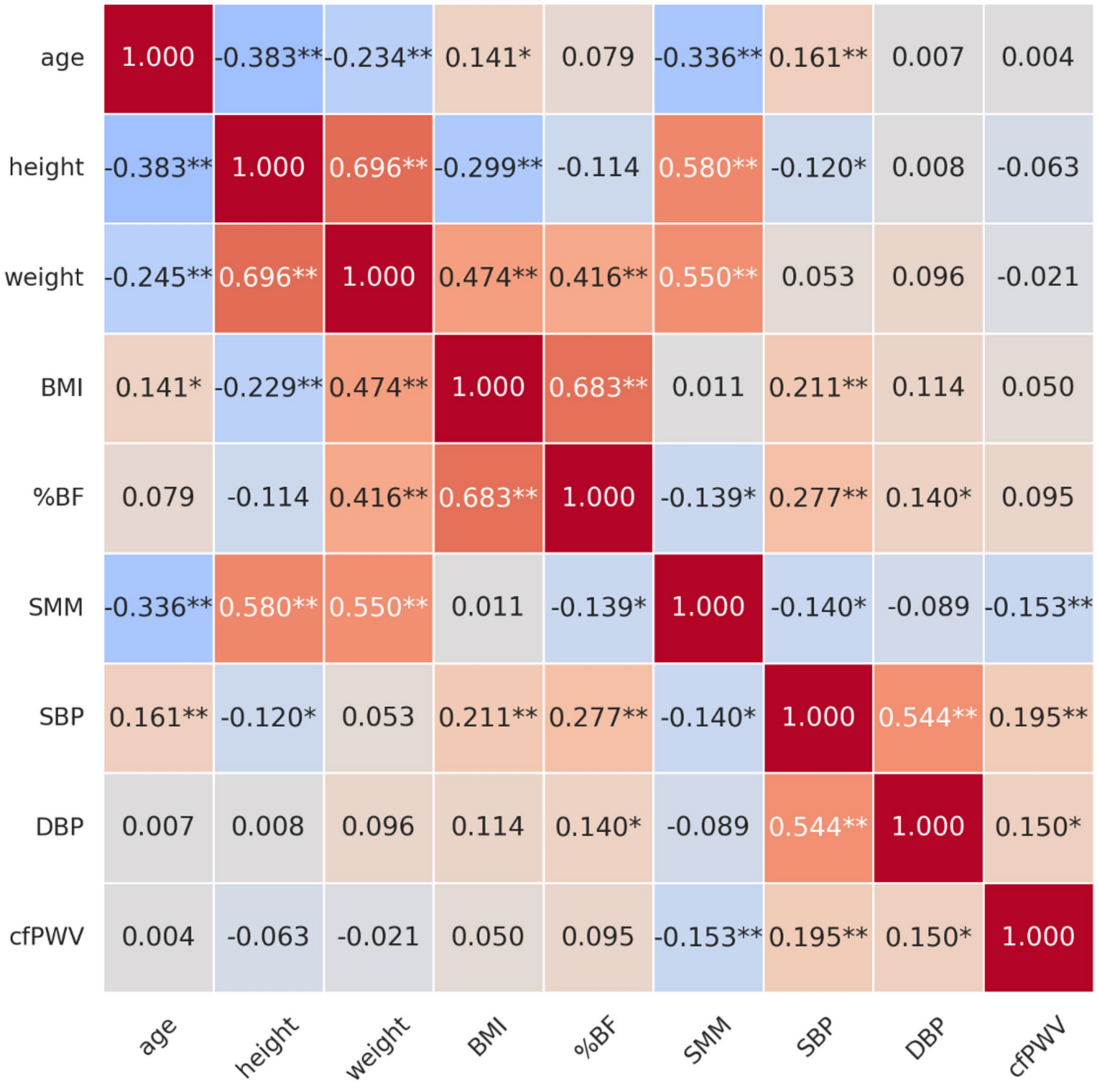
Correlation between age, body composition, blood pressure, and arterial stiffness in obese older women. *p < 0.05, **p < 0.01. BMI, body mass index; %BF, percentage of body fat; SMM, skeletal muscle mass; SBP, systolic blood pressure; DBP, diastolic blood pressure; cfPWV, carotid-femoral pulse wave velocity.

Figure 1 illustrates the correlations among age, body composition variables, blood pressure (BP), and arterial stiffness (AS), as measured by carotid-femoral pulse wave velocity (cfPWV), in obese older women.

### Correlation with age

A significantly negative correlation was identified between age and height (*r* = −0.383, *p* < 0.01), indicating that the height tended to decrease with increasing age. The results indicated a substantially negative correlation between age and SMM (*r* = −0.336, *p* < 0.01), suggesting that as age increased, SMM levels tended to decline. In contrast, a significantly positive correlation was observed between the age and BMI (*r* = 0.141, *p* < 0.05), suggesting that BMI tended to increase with age. A significantly positive correlation was also identified between age and SBP (*r* = 0.161, *p* < 0.01), implying that older individuals were more likely to have elevated SBP.

### Correlation with height

A significantly positive correlation was identified between height and weight (*r* = 0.696, *p* < 0.01), suggesting that taller individuals tend to weigh more. Additionally, a robust positive correlation was observed between height and SMM (*r* = 0.580, *p* < 0.01), suggesting that taller individuals tended to have higher SMM levels. Conversely, BMI and height (*r* = −0.299, *p* < 0.01) demonstrated a substantially negative correlation, indicating that taller individuals generally had a lower BMI.

### Correlation with weight

A significantly positive correlation was observed between body weight and BMI (*r* = 0.474, *p* < 0.01), %BF (*r* = 0.416, *p* < 0.01), and SMM (*r* = 0.550, *p* < 0.01). This implies that as weight increases, BMI, %BF, and SMM tend to increase as well.

### Correlation with BMI

A significantly positive correlation was detected between BMI and %BF (*r* = 0.683, *p* < 0.01). As BMI increases, %BF also increases. The results also demonstrated a significantly positive correlation of BMI with SBP (*r* = 0.211, *p* < 0.01), but not with SMM.

### Correlation with %BF

%BF was positively correlated with SBP (*r* = 0.277, *p* < 0.01), diastolic blood pressure (DBP) (*r* = 0.140, *p* < 0.05), and negatively correlated with SMM (*r* = −0.139, *p* < 0.05), indicating that individuals with higher body fat tend to have increased BP and lower muscle mass.

### Correlation with SMM

SMM was negatively correlated with age (*r* = −0.336, *p* < 0.01), %BF (*r* = −0.139, *p* < 0.05), cfPWV (*r* = −0.153, *p* < 0.05), and SBP (*r* = −0.140, *p* < 0.05). These findings suggest that greater muscle mass may be protective, contributing to lower AS and BP levels.

### Correlation with blood pressure

SBP exhibited a significantly positive correlation with %BF (*r* = 0.277, *p* < 0.01), BMI (*r* = 0.211, *p* < 0.01), and cfPWV (*r* = 0.195, *p* < 0.01), a measure of AS. This suggests that AS is likely to increase with increasing SBP. Diastolic blood pressure (DBP) demonstrated a significantly positive correlation with both %BF (*r* = 0.140, *p* < 0.05) and SBP (*r* = 0.544, *p* < 0.01), as well as a significantly positive correlation with cfPWV (*r* = 0.150, *p* < 0.05). Therefore, elevated DBP is associated with increased %BF.

### Correlation with cfPWV

A significantly positive correlation was identified between cfPWV and both SBP (*r* = 0.195, *p* < 0.01) and DBP (*r* = 0.150, *p* < 0.05), indicating that AS increases with rising SBP and DBP. However, a substantially negative correlation was observed between cfPWV and SMM (*r* = −0.153, *p* < 0.05), suggesting that higher SMM levels were associated with reduced AS.

### Group differences by physical activity level

#### Body composition

Table 2 shows the differences in the body composition according to levels of PA. BMI and %BF exhibited significant disparities based on levels of PA. BMI (*t* = 3.705, *p* < 0.001) was significantly higher in the inactive group (26.27 ± 2.11 kg/m^2^) than in the active group (25.50 ± 1.29 kg/m^2^), suggesting that older women who are less physically active are more likely to have an elevated BMI. Furthermore, %BF (*t* = 5.605, *p* < 0.001) was significantly higher in the inactive group (37.39 ± 2.57%) than in the active group (36.02 ± 1.32%), suggesting a close association between the level of PA and %BF. SMM (*t* = −3.802, *p* < 0.05) was significantly higher in the active group (20.99 ± 1.80 kg) compared to the inactive group (20.05 ± 2.35 kg), indicating that physically active older women may maintain more muscle mass. The differences in height and weight by PA were not statistically significant. These results suggest that PA may have a positive impact on reducing BMI and %BF and increasing SMM.

**Table 2:** Differences in body composition according to physical activity levels.

Variables	ING	AG	*t*-value	*p*-value
Height (cm)	150.71 ± 6.42	151.89 ± 6.09	−1.604	0.110
Weight (kg)	59.66 ± 5.93	58.85 ± 4.70	1.292	0.197
BMI (kg/m^2^)	26.27 ± 2.11	25.50 ± 1.29	3.705	0.000***
Body fat (%)	37.39 ± 2.57	36.02 ± 1.32	5.605	0.000***
SMM (kg)	20.05 ± 2.35	20.99 ± 1.80	−3.802	0.011*

Values are presented as mean (M) ± standard deviation (SD). *p < 0.05, ***p < 0.001.

ING, inactive group; AG, active group.

#### Blood pressure

**Figure 2:**
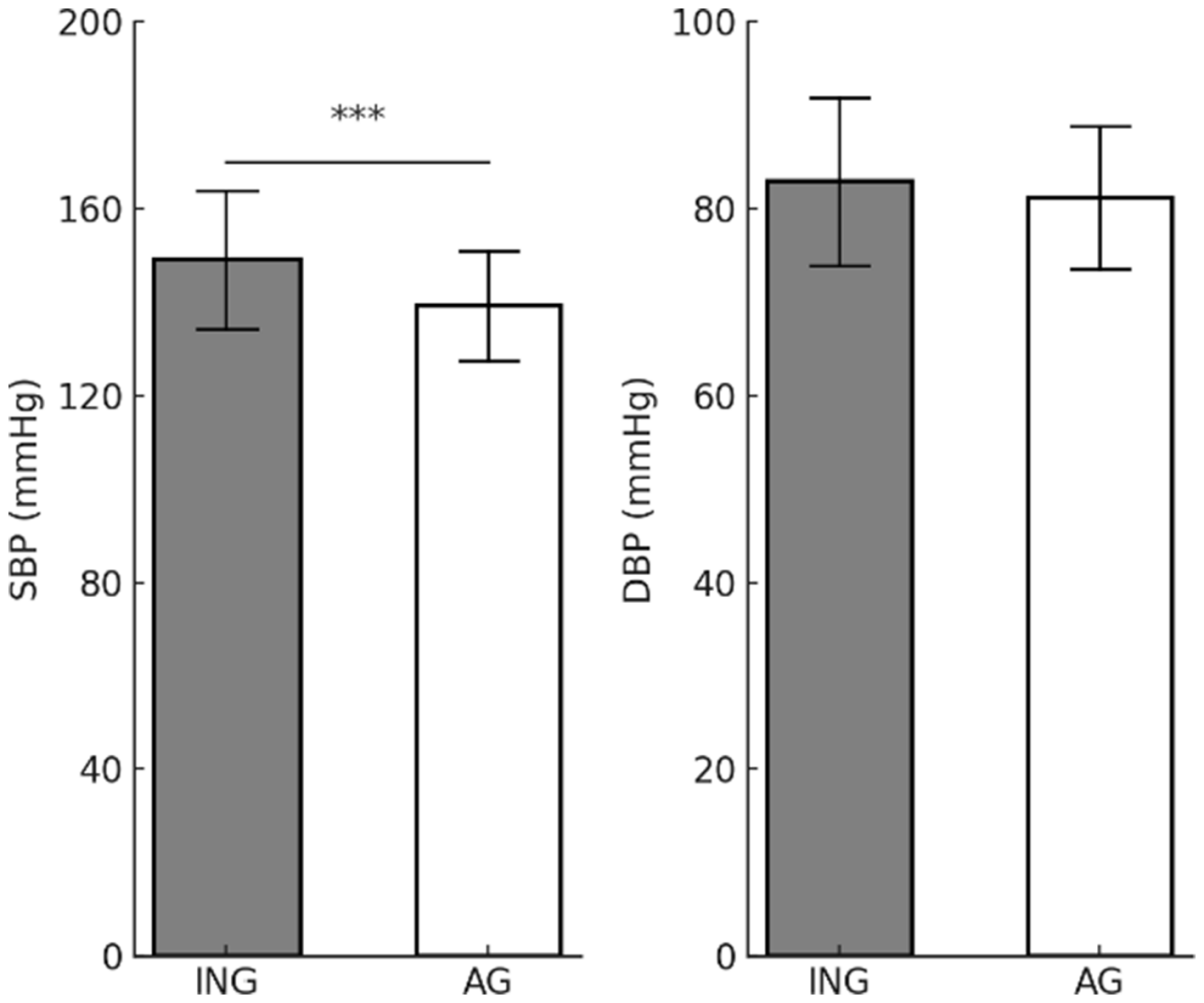
Differences in blood pressure according to physical activity levels. Values are presented as mean (M) ± standard deviation (SD). ***p < 0.001. SBP, systolic blood pressure; DBP, diastolic blood pressure; ING, inactive group; AG, active group.

Figure 2 shows the differences in BP according to levels of PA. The analysis revealed significant disparities in the SBP according to levels of PA; however, no substantial variations in the DBP were observed. SBP (*t* = 6.293, *p* < 0.001) was found to be significantly higher in the inactive group (149.08 ± 14.82 mmHg) compared to the active group (139.12 ± 11.83 mmHg), indicating that PA may potentially reduce SBP. In contrast, DBP (*t* = 1.744, *p* = 0.082) did not differ significantly between the inactive (82.89 ± 8.99 mmHg) and active groups (81.17 ± 7.63 mmHg). These results imply that PA in older women is more effective in controlling primarily SBP. Further investigation is necessary to determine whether long-term PA interventions can also affect DBP.

#### Arterial stiffness

**Figure 3:**
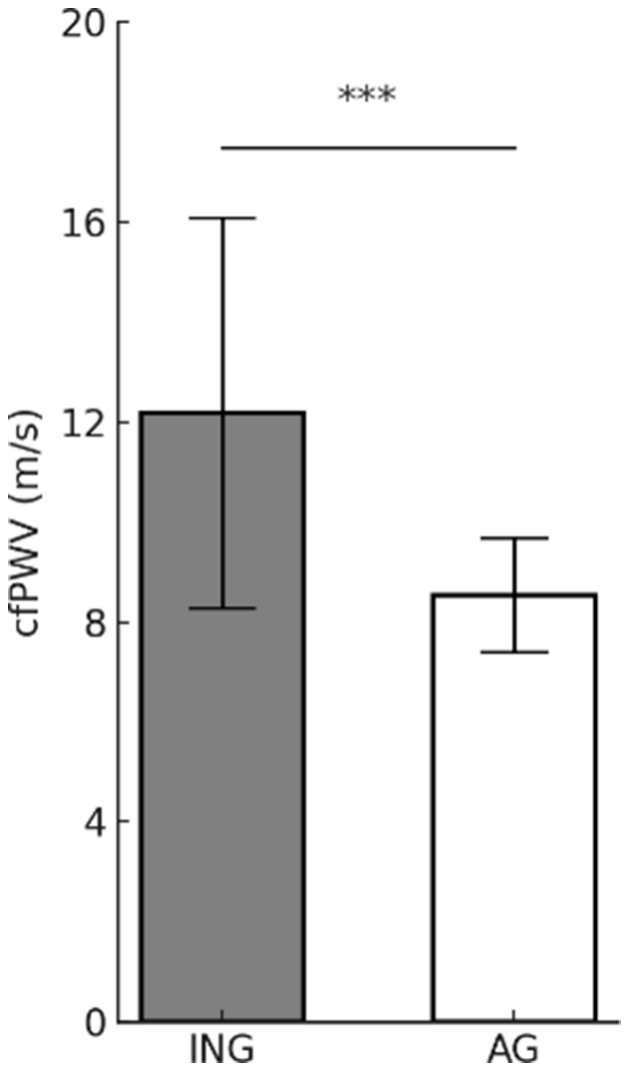
Differences in arterial stiffness according to physical activity levels. Values are presented as mean (M) ± standard deviation (SD). ***p < 0.001. cfPWV, carotid-femoral pulse wave velocity; ING, inactive group; AG, active group.

Figure 3 displays the difference in cfPWV according to levels of PA. The cfPWV is a key indicator of AS. The inactive group (12.18 ± 3.90 m/s) exhibited significantly higher cfPWV (*t* = 4.765, *p* < 0.001) values compared to the active group (8.54 ± 1.14 m/s), suggesting a potentially positive effect of PA in reducing AS.

Consequently, physically active women are likely to have better cardiovascular health due to lower AS compared to inactive older women.

This finding aligns with those of previous studies, suggesting that regular PA can contribute to maintaining the elasticity of arteries and reducing the risk of developing CVD.

## Discussion

This study sought to elucidate the associations between levels of PA and key health indicators specifically body composition, BP, and AS in obese older women. Our findings revealed that higher PA levels were associated with more favorable health profiles, including lower BMI, lower %BF, higher SMM, reduced SBP, and decreased cfPWV, a marker of AS.

### Correlations among anthropometric and physiological variables

This study analyzed the correlations between height, weight, BMI, %BF, SMM, and BP, and found that the height was positively correlated with both the body weight and SMM, while height and BMI demonstrated a significantly negative correlation. This suggests that taller individuals tend to have higher body weight and SMM but a lower BMI. These results are consistent with those of a previous study (36) that reported that taller people have more SMM and that muscle mass is a major contributor to weight gain. These findings also align with those in the existing research (37) indicating that taller individuals tend to have a comparatively lower BMI, as BMI is calculated as the ratio of height to weight.

A significantly positive correlation was observed between body weight and BMI, %BF, and SMM. This indicates that as the weight increases, there is a tendency for an increase in SMM as well as body fat mass. Thus, weight gain may be influenced not only by obesity but also by muscle mass gain. These results are consistent with the findings of Cho et al., who suggested that weight gain is associated with increased body fat as well as increased muscle mass (38).

A significantly positive correlation was identified between BMI and %BF, suggesting that as the BMI increased, the %BF tended to rise concomitantly. Therefore, BMI can serve as a reliable predictor of the body fat mass. This is consistent with the findings of previous studies (39, 40) reporting a strong correlation between the BMI and %BF. However, the BMI did not demonstrate a statistically significant correlation with the SMM, indicating that BMI is more closely associated with %BF than with SMM. These results are consistent with the findings of Wang et al., who reported that the BMI exhibits a stronger correlation with %BF than with the muscle mass (41).

BMI also showed a significantly positive correlation with the SBP. This implies that individuals with a BMI categorizing them as overweight or obese are more prone to elevated BP, which aligns with the conclusions of previous studies (42, 43) that identified obesity as a significant contributing factor to hypertension.

A significantly positive correlation was found between %BF and SBP, in line with the conclusions of previous studies that demonstrated a positive correlation between higher %BF and hypertension. This observation supports the established relationship between obesity and hypertension in the literature (44). Increased %BF has been linked to visceral fat accumulation. Given the adverse effects of visceral fat on vascular health (45), it can be concluded that managing %BF is vital for maintaining cardiovascular health.

This study employed a quantitative approach to examine the correlations among body composition indices, thereby offering a deeper understanding of the impact of the relationship between body weight and %BF on health-related indicators, such as BP. The correlation between %BF and BP indicates that the regulation of %BF may constitute a more efficacious health management strategy than merely monitoring weight. Future research should involve comprehensive analyses of diverse populations. Moreover, longitudinal investigations are imperative to elucidate the health implications of body composition changes.

### Effects of physical activity on body composition

Reduced PA among older individuals may increase the probability of obesity (46), owing to a decrease in the muscle mass and increase in the body fat mass. A decrease in muscle mass can result in diminished physical strength and motor function. This, in turn, can reduce the frequency of PA among older individuals (47), which can have a detrimental effect on their capacity to perform activities of daily living (48).

Obesity caused by increased body fat has been identified as a significant risk factor for the development of metabolic syndrome and atherosclerosis (49), and it can increase the risk of death from cardiovascular disease (50). However, reducing sedentary time and increasing PA have been shown to be effective strategies for improving obesity-related metabolic disorders (51), and lowering the risk of cardiovascular disease (52).

Our previous study demonstrated improvements in body composition among older women through regular participation in PA (53). Similarly, a study by Morio et al. reported a decrease in body fat and increase in muscle mass in older individuals with a mean age of 62.8 years who engaged in regular PA (54). This has been suggested to result from enhanced muscle metabolism (55) and increased fat oxidation through PA, leading to a reduction in body fat (56).

Our results revealed a significantly lower %BF and BMI in the active group compared to the inactive group and a significantly higher SMM in the active group compared to the inactive group, consistent with the findings of previous studies. Furthermore, age and weight were negatively correlated with the SMM and positively correlated with the BMI. These findings suggest that PA has the potential to positively impact BMI and body fat in obese older individuals and may help prevent age-related loss of muscle mass. Therefore, PA may provide multiple health benefits, including the prevention and amelioration of obesity and related conditions in older individuals.

### Effects of physical activity on blood pressure

Obesity is a risk factor for the development of hypertension (57), and weight gain is known to increase the risk of hypertension by approximately 65–75% (43). It has been reported that over 70% of individuals with hypertension are overweight or obese (58).

A previous study that compared obese and normal-weight individuals reported a higher prevalence of hypertension in obese individuals than in normal-weight individuals (59). Hypertension, in turn, increases the risk of cardiovascular disease and mortality (60).

The development of hypertension in obese individuals has been attributed to several factors, including inflammation, leptin, and activation of the renin-angiotensin and sympathetic nervous systems (61–63). PA is suggested to lower blood pressure in overweight or obese individuals (64) by reducing friction between blood and vessel wall (65), suppressing sympathetic nervous system activity, and enhancing the release of vasodilators (66).

Ishikawa et al. reported that a weekly total of more than 60 min of PA led to a reduction in SBP that was greater than the reduction achieved through 30–60 min of PA per week (67).

The findings of this study demonstrated that SBP was significantly lower in the active group compared to the inactive group, thereby corroborating the findings of previous studies.

Furthermore, this study showed that age, BMI, and %BF correlated with the SBP, which aligns with the findings of previous studies (68). Consequently, participation in regular PA and improvement in BMI and body fat in obese older individuals may have a positive impact on BP control. Therefore, engaging in PA may be an effective approach for both the prevention and management of hypertension.

### Effects of physical activity on arterial stiffness

Increased body fat and obesity, often resulting from physical inactivity, may induce damage to the vascular endothelial cells and increase inflammation. This, in turn, precipitates diminished vascular elasticity and augmented arterial pressure, consequently giving rise to cardiovascular disorders such as hypertension and atherosclerosis (69, 70). Furthermore, the obesity-induced progression of vascular damage and fibrosis leads to increased AS as the elasticity and cushioning capacity of the arteries decrease (71).

Previous studies have revealed a relationship between obesity and AS (72), and a correlation between high PWV and CVD (73, 74).

Importantly, regular PA has been demonstrated to have a direct effect on improving vascular function and structure (75), reducing %BP, and improving AS (76).

Ahmadi et al. reported that reducing sedentary behavior and engaging in regular PA could slow the progression of AS (77). Similarly, Tanaka et al. suggested that PA improves AS in older adults (78).

It is well-documented that the increased blood flow induced by PA exerts a positive influence on vascular endothelial cells, leading to improved AS. The underlying mechanism involves structural and functional changes in the vessel wall (75), including the enhanced production and bioavailability of NO (79).

This study found that cfPWV levels were significantly lower in the active group compared to the inactive group and that cfPWV was associated with the SBP and SMM. These findings suggest that PA-induced reduction in the BP and increase in SMM may lead to an improvement in the AS. These findings provide evidence that PA is an important strategic intervention for the prevention and improvement of cardiovascular disease in obese older individuals.

Despite the strengths of this study, several limitations should be acknowledged. First, the sample was limited to obese women aged 65 and older, which limits the generalizability of the findings to other age groups and males. Second, this was a cross-sectional study, preventing causal inference. Longitudinal studies are needed to establish temporal relationships and the long-term effects of PA on cardiovascular and metabolic health. Third, although accelerometry provided objective data on PA intensity and duration, we did not collect data using the International Physical Activity Questionnaire (IPAQ). As a result, we were unable to categorize PA by type (e.g., endurance, resistance), frequency, or specific duration. Fourth, potential confounding lifestyle variables such as dietary intake, medication use, and sleep patterns were not controlled. Fifth, the waist-to-hip ratio, a recognized marker of visceral fat accumulation and cardiovascular disease risk, was not assessed in this study. Future studies should incorporate these variables to better understand the mechanisms underlying PA-related health outcomes.

## Conclusion

This study examined the relationships between PA levels and health outcomes including body composition, BP, and AS in obese older women. The results demonstrated significant positive correlations between %BF and SBP, and between SBP and cfPWV, suggesting that obesity-related factors contribute to elevated BP and increased AS. Compared to inactive participants, the physically active group exhibited significantly lower BMI, %BF, and cfPWV, and significantly higher SMM. These findings highlight the potential of regular PA to ameliorate obesity-related health risks and support vascular health in aging populations.

Future research should explore the effects of different PA intensities, frequencies, and durations on cardiometabolic outcomes. Long-term intervention studies are also warranted to determine the sustained effects of PA on body composition and cardiovascular function in diverse aging populations.

## Data Availability

The original contributions presented in the study are included in the article/supplementary material, further inquiries can be directed to the corresponding author.
